# Effect of Charged Block Length Mismatch on Double Diblock Polyelectrolyte Complex Micelle Cores

**DOI:** 10.1021/acs.macromol.3c00555

**Published:** 2023-07-06

**Authors:** Kaden C. Stevens, Alexander E. Marras, Trinity R. Campagna, Jeffrey M. Ting, Matthew V. Tirrell

**Affiliations:** † Pritzker School of Molecular Engineering, The University of Chicago, Chicago, Illinois 60637, United States; ‡ Walker Department of Mechanical Engineering, The University of Texas at Austin, Austin, Texas 78712, United States; § Texas Materials Institute, The University of Texas at Austin, Austin, Texas 78712, United States; ∥ Nanite Inc., Boston, Massachusetts 02109, United States

## Abstract

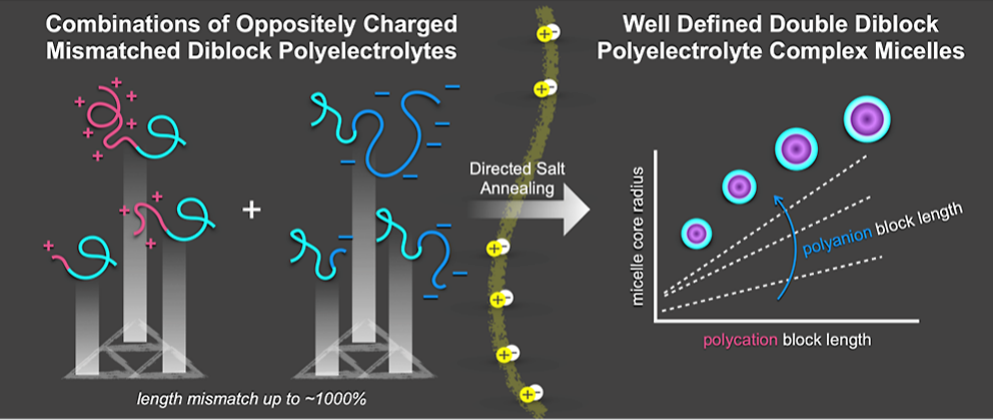

Polyelectrolyte complex micelles are hydrophilic nanoparticles that self-assemble in aqueous environments due to associative microphase separation between oppositely charged blocky polyelectrolytes. In this work, we employ a suite of physical characterization tools to examine the effect of charged block length mismatch on the equilibrium structure of double diblock polyelectrolyte complex micelles (D-PCMs) by mixing a diverse library of peptide and synthetic charged-neutral block polyelectrolytes with a wide range of charged block lengths (25–200 units) and chemistries. Early work on D-PCMs suggested that this class of micelles can only be formed from blocky polyelectrolytes with identical charged block lengths, a phenomenon referred to as chain length recognition. Here, we use salt annealing to create PCMs at equilibrium, which shows that chain length recognition, a longstanding hurdle to repeatable self-assembly from mismatched polyelectrolytes, can be overcome. Interestingly, D-PCM structure–property relationships display a range of values that vary systematically with the charged block lengths and chemical identity of constituent polyelectrolyte pairings and cannot be described by generalizable scaling laws. We discuss the interdependent growth behavior of the radius, ionic pair aggregation number, and density in the micelle core for three chemically distinct diblock pairings and suggest a potential physical mechanism that leads to this unique behavior. By comparing the results of these D-PCMs to the scaling laws recently developed for single diblock polyelectrolyte complex micelles (S-PCMs: diblock + homopolymer), we observe that D-PCM design schemes reduce the size and aggregation number and restrict their growth to a function of charged block length relative to S-PCMs. Understanding these favorable attributes enables more predictive use of a wider array of charged molecular building blocks to anticipate and control macroscopic properties of micelles spanning countless storage and delivery applications.

## Introduction

Understanding the self-assembly of polyelectrolytes remains an outstanding problem in polymer science with widespread implications.^1,2^ The ubiquity of ion-containing biopolymers with remarkably complex structures has long fascinated polymer scientists seeking to emulate the intricate self-assembly that enables cellular compartmentalization, enzymatic catalysis, and viral delivery.^2^ Unfortunately, in many situations, biomimicry remains either impractical or unattainable in scope, so polymer scientists often focus on developing synthetic systems that interface with biology instead.^3^ To this end, a great deal of effort has been devoted to developing nanocarriers to deliver therapeutics in a variety of clinical applications.^4,5^ The most common approach has been the utilization of amphiphilic molecules. With judicious placement, the hydrophobic groups of these molecules can self-assemble in aqueous environments to form hydrophobic domains capable of sequestering hydrophobic cargo. While successful in certain instances, amphiphilic assembly can require complex protocols or materials to encapsulate hydrophilic therapeutics and may suffer from poor circulation or toxicity, motivating the development of alternative approaches to biomaterials.^3^ A more complete understanding of polyelectrolyte assemblies could address key knowledge gaps and enable the development of next generation biomaterials.

Polyelectrolyte complexes (PECs) stand out as a promising biomaterials platform since a host of therapeutic molecules are charge-bearing, and many adverse biological interactions are known or suspected to be mediated by charge–charge interactions.^6,7^ PECs form spontaneously upon the mixture of oppositely charged polyelectrolytes due to associations between oppositely charged polyelectrolytes and the entropic gain resulting from the release of small molecule counterions, resulting in a hydrated polymer-rich phase.^8−13^ If desired, blocky and non-linear architectures can be incorporated to induce phase separation at smaller scales, resulting in ordered gels and nanoparticles with PEC domains. Among these structured PEC materials, polyelectrolyte complex micelles (PCMs) have gained particular interest for their ability to partition hydrophilic biomolecules such as nucleic acids (e.g., new DNA or RNA genes, oligonucleotide gene modulators, or gene editors) or charged proteins within core–shell nanoparticles aiding protection, shielding, and biodistribution.^14−17^ PCMs were pioneered by Harada and Kataoka in the 1990s, and since that time single diblock (AB + C) and double diblock (AB + AC) PCMs have emerged as the two primary micelle formulations.^18−21^ As the names suggest, single diblock PCMs (S-PCMs) employ one diblock polyelectrolyte (with a neutral A block and charged B block) complexing with an oppositely charged homopolyelectrolyte (C block) to create core–shell nanostructures,^3,18,22−25^ whereas double diblock PCMs (D-PCMs) utilize two oppositely charged AB and AC diblocks.^20,26−33^

The differences between single and D-PCMs may seem trivial, but the covalent attachment of charged and neutral blocks in every constituent polyelectrolyte establishes the most important structural interplay for D-PCM structure: namely, the competition between charge neutralization within the core and strict core–shell segregation.^34,35^ This interplay results in D-PCMs having smaller particle size, lower resistance to dissolution by salt, and smaller aggregation numbers than comparable S-PCMs.^21,34,35^ Fascinating studies of the fundamental assembly properties of D-PCMs have demonstrated unique behavior such as homogeneous mixing of immiscible corona forming blocks and homopolymer displacement from S-PCMs to favor D-PCMs. These studies suggest that a complex interplay of thermodynamic properties distinguish S-PCMs from D-PCMs and have prompted the exploration of these materials in a variety of storage and delivery applications.

**Figure 1:**
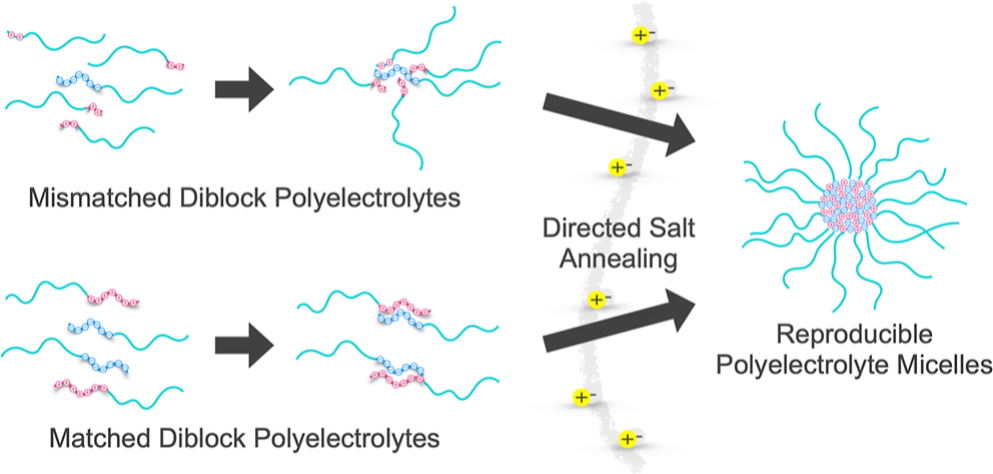
Schematic model for the formation of mismatched and matched PCMs through salt annealing.

Within the growing literature on double-diblock PEC nanostructures, few have examined the effect of blending two diblocks with mismatched charged block lengths, despite the potential understanding and utility that unmatched block lengths could provide. In seminal work on D-PCMs, Harada and Kataoka reported that when mismatched charged length diblock polyelectrolytes were mixed, no PCMs formed.^36^ Instead, small aggregates were formed. The same group concluded that the aggregates are “unit polyionic complexes” by calculating their molecular weight using light scattering.^35^ These small aggregates were hypothesized to be composed of one long diblock compensated by enough short diblocks to neutralize charge (Figure 1). The neutral length of all polymers was the same. These unit polyionic complexes were theorized to be an intermediate stage of PCM formation that were unable to proceed to well-defined core shell structures due to an inability to reconcile the need for charge compensation and core–shell segregation. Chain length recognition, the term used to describe the unique ability of matched diblocks to grow past small aggregates and form uniform D-PCMs, shaped many experimental studies that followed. Since the publication of these results, a majority of D-PCM studies focused on polyelectrolyte pairs with less than 10% difference in oppositely charged block length. To accommodate the restrictions of chain length recognition, some works make structurally identical but oppositely charged polyelectrolytes by using reactive precursor polymers that are subsequently functionalized using click chemistry.^28−30,37,38^ Overall, this result has been hugely consequential in the design of ordered PEC structures from gels to PCMs, but recent results in PCM dynamics lead us to question some foundational assumptions behind chain length recognition.

Some assume that since PCMs form spontaneously upon mixing, they rapidly reach thermodynamic equilibrium, and, thus, the failure of mismatched diblocks to form PCMs is due to poor chain length recognition and not kinetics.^36^ However, this interpretation is at odds with recent chain exchange measurements in PCMs that suggest large energetic barriers to molecular rearrangements in low salt environments, as well as studies of micelle formation that demonstrate the role of kinetic barriers in PCM formation and equilibration.^26,39−41^ Furthermore, our group has shown that PCMs formed by rapidly mixing polyelectrolyte solutions do not form PCMs with consistent and reproducible structures. Taken together, these studies confirm that many PCMs are not at equilibrium on the timescale of experiment, despite forming spontaneously in water.^15^ To circumvent this issue, our group developed annealing protocols that gave PCMs the necessary mobility to rearrange and reach their minimum energy states, leading to reproducible PCMs.^15,42,43^ In this work, we use directed salt annealing to abandon the limitations of chain length recognition and explore a wide ratio of diblock polyelectrolytes with mismatched charged block lengths and discover unique interdependent trends as a function of charged block length in the core of D-PCMs (Figure 1).

## Results and Discussion

**Scheme 1:**
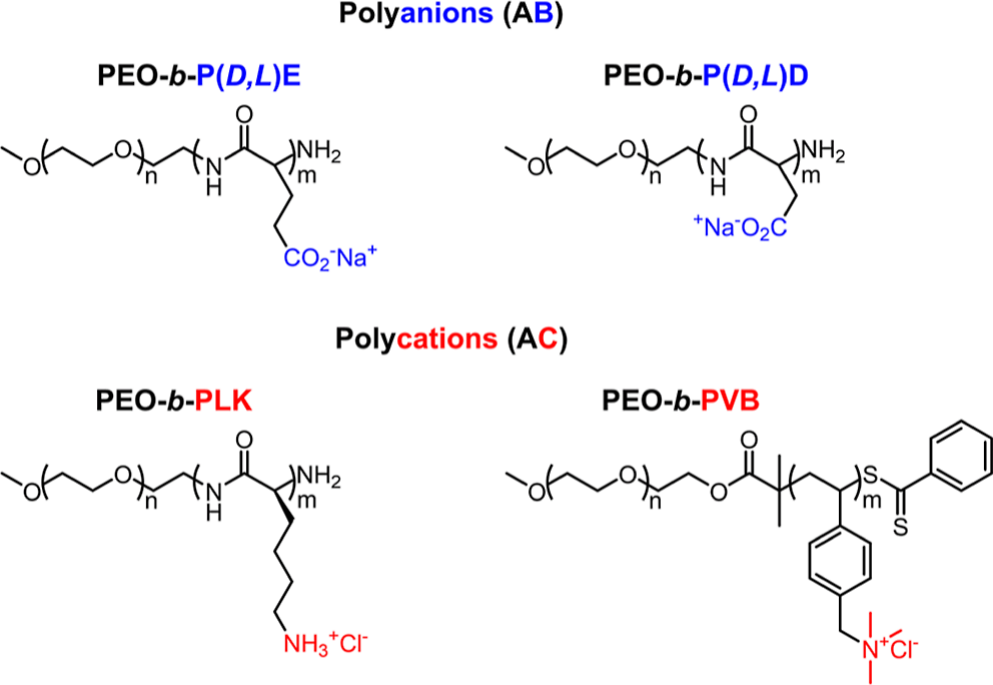
Chemical Structures of Polyelectrolytes Examined in This work: PEO-*b*-P(d,l)D (D).PEO-*b*-P(d,l)E (E), PEO-*b*-P*L*K (K), and PEO-*b*-PVB (V)

To understand the influence of charged block length mismatch on D-PCMs, we created a library of diblock polyelectrolytes with poly(ethylene oxide) (PEO) as the neutral block and poly(glutamic acid) (P(d,l)E), poly(aspartic acid) (P(d,l)D), poly(lysine) (P*L*K), and poly((vinylbenzyl)trimethylammonium chloride) (PVB) as the charged blocks (Scheme 1). We chose to study diblock polymers with a consistent neutral block length of approximately 5 kg/mol PEO and charged block lengths of 25–200 units to examine charged block lengths that spanned an order of magnitude and are within the typical size range for PCM studies.^44^ To refer to data concisely, we use the shorthand of E, D, K, and V to refer to PEO-*b*-P(d,l)E, PEO-*b*-P(d,l)D, PEO-*b*-P*L*K, and PEO-*b*-PVB, respectively. For example, micelles composed of combinations of PEO-*b*-P*L*K and PEO-*b*-P(d,l)D will be referred to as KD pairings for brevity. In addition, the term set block length is used throughout the discussion to refer to the charged block length that is held constant within a data set.

Previous work in our group has shown that the dominant form of micelle preparation, rapid mixing, can lead to kinetically trapped PCMs, leading to inconsistent micelle structure.^15^ To address this issue, we developed salt-annealing protocols for PCMs where the oppositely charged polymers are prepared in a high salt environment and then dialyzed to the final salt concentration over the course of days. This method ensures that the micelles have ample freedom to rearrange and avoid kinetic restrictions and has been shown to produce thermodynamically stable, reproducible PCMs.^15,43^ To prepare our PCMs, diblock polyelectrolytes were first mixed at a 1:1 charge ratio at an ionic strength high enough to minimize complexation of PCMs and then slowly dialyzed to the final buffer concentration of 50 mM NaCl in 10 mM HEPES.

To fully characterize the shape, structural features, and aggregation number of our PCMs, we use a combination of small-angle X-ray scattering (SAXS), dynamic light scattering (DLS), and cryogenic transmission electron microscopy (cryo-TEM). Most diblock polyelectrolyte pairings assemble into well-defined spheroidal core–shell micelles regardless of charged block length symmetry. However, KE pairings do not form monodisperse spherical PCMs, but instead form aggregates with rodlike structures according to SAXS, possibly due to the formation of beta sheets in the PCM core.^45^ These ineffective pairings represent a small minority of the total polyelectrolyte pairs studied and were not included in our analysis.

**Figure 2:**
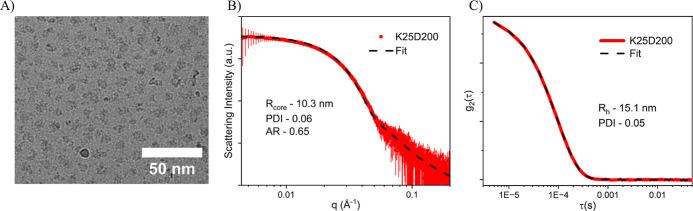
PCM characterization (A) cryo-TEM images of the PCMs formed by mixing 5kPEG-*b*-P*L*K_25_ and 5kPEG-*b*-P(d,l)D_200_ (K25D200). Cryo-TEM shows PCM cores but is unable to resolve PCM coronas due to the low contrast between PEG and water. Scale bar = 50 nm. (B) Experimental SAXS data and fit used to determine the PCM core radius *R*_core_, PDI, and AR for K25D200. (C) DLS and fit provide total particle size through *R*_h_, which can be used to calculate PCM shell thickness (*H* = *R*_h_– *R*_core_).

The remaining PCMs formed compact, spheroidal nanoparticles as visualized by a representative TEM image in Figure 2A. As detailed in Table S1, the scattering contrast between the core-forming polyelectrolyte repeat units and water is an order of magnitude stronger than that of PEO. As a result, SAXS patterns of these systems are dominated by scattering contributions from the PCM core, as has been shown previously.^15,42,43,46,47^ Our collected SAXS data was then fit with a spheroidal form factor to model the PCM core supplemented with a high q power law fit to account for scattering from neutralized polymer chains within the core.^15,42,43^ The data and model fits show excellent agreement, as shown in Figures 2B and S3. A majority of PCM fits had core radii values that ranged from 9–25 nm with aspect ratios (ARs) from 0.5–1.0 and narrow polydispersity indexes (PDIs). To measure the hydrodynamic radius of our PCMs, we utilized DLS and obtained sizes of 10–60 nm. Figure 2C shows the fitting of a representative autocorrelation function. Combining SAXS and DLS allows us to calculate the corona thickness by subtracting the core radius from the hydrodynamic radius, resulting in shell thicknesses that mostly ranged from 4–15 nm. A detailed list of complete PCM features can be found in Table S2.

### Core Growth

To investigate the relationship between charged block length mismatch and PCM structural features, we first looked at the relationship between total charged block length and PCM core radius. Intuitively, one might expect that a micelle feature like core radius would scale with the sum of the charged block lengths of the constituent polyelectrolytes, as both charged blocks contribute to core size. However, there is a poor relationship between the total charged block length and core size (Figure S4). Instead, we grouped our PCMs into sets where one charged block length is held constant as the oppositely charged block length is varied.

**Figure 3:**
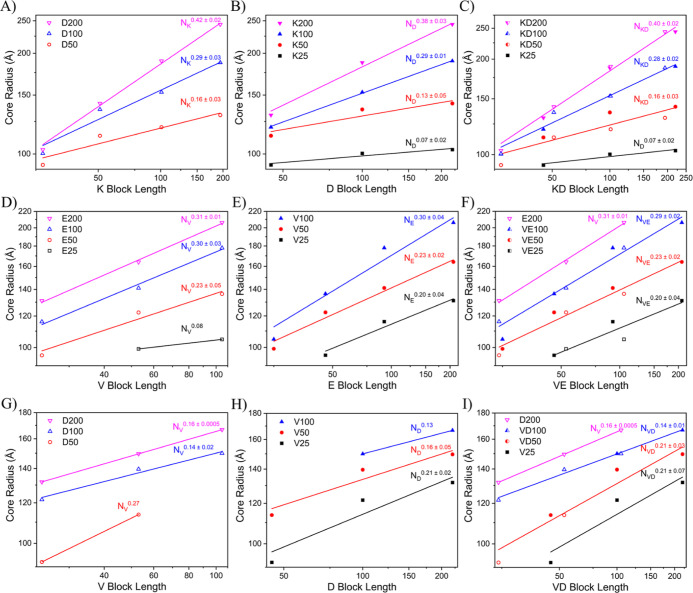
Core radius vs block length determined by fitting SAXS data and plotting the results against charged block length. A power law fit was applied to each data set, which are divided into separate colors/symbols. Every diblock polyelectrolyte has a neutral PEO block length of ∼5k. Charged block length names are abbreviated from P*L*K to K, PVB to V, P(d,l)D to D, and P(d,l)E to E for brevity. Across all datasets, the datapoints/fits are represented by colors, with black representing DP ∼25, red symbols representing DP ∼50, blue symbols representing DP ∼100, and pink symbols representing DP ∼200. Open datapoints signify an anionic frame of reference, where the data are grouped into sets of constant anionic block length and plotted against varying cationic block length. Filled data signify a cationic frame of reference, where data are grouped into sets of constant cationic block length and plotted against varying anionic block lengths. Half-filled data signify a composite dataset where both anionic and cationic frames of reference are represented in the same color and are fit together. (A–C) Show the KD pairing. (A) and (B) show the distinct frames of reference and in (C) both datasets are overlaid to remove any preference for one diblock when fitting the results. (D–F) and (G–I) repeat the same graphing pattern for VE and VD polyelectrolyte pairings.

Since each of these micelles is composed of two diblock polyelectrolytes (AB + AC), there are two ways to examine each set of data: either a fixed AB polymer length and variable AC length, or the converse. For example, in Figure 3A, we plot the core radius results of the KD dataset in the frame of reference holding the block lengths of PEO-*b*-P(d,l)D constant at D50 (open red), D100 (open blue), and D200 (open pink) and varying P*L*K block lengths from 25 to 200. Figure 3B represents the same data visualized with closed symbols by holding PEO-*b*-P*L*K block length constant at K25 (black), K50 (red), K100 (blue), and K200 (pink) and varying P(d,l)D block length from 50 to 200. Both perspectives of the data were fit with a power law, as shown in the text labels of each plot. To remove bias toward one polyelectrolyte, we also consolidated the datasets from the left two columns of Figure 3 into the right column (Figure 3C,F,I), such that in Figure 3C, the KD50 dataset is a composite of the D50 set from Figure 3A (open symbols) and the K50 set from Figure 3B (closed symbols). This gives a chemically agnostic description of the growth of the PCM cores without biasing toward one polyelectrolyte and allows us to evaluate the influence of diblock mismatch.

### Interdependent Core Growth

Our data show that larger charged block lengths lead to increased core size in all sets regardless of polyelectrolyte pairing. However, unlike single diblock PCMs, the different sets cannot be normalized and combined into a single predictive scaling law because the exponential value of the power law fits varies as a function of set block length. For example, in the KD system, the power law fit applied to the sets grows from 0.07 ± 0.02 to 0.40 ± 0.02 as the set block length grows from 25 to 200. The systematically increasing growth rate as a function of set block length suggests that AB + AC micelle cores grow via a uniquely interdependent mechanism, whereby the size of AB polyelectrolytes modulate the ability of AC polyelectrolytes to contribute to core growth.

To the best of our knowledge, this interdependent behavior has not been observed in uncharged AB + AC amphiphilic systems. Furthermore, interdependent growth in uncharged systems may prove extremely difficult to observe due to the tendency of chemically dissimilar neutral polymers to undergo macrophase separation upon mixing. PCMs, on the other hand, exhibit dynamic, uniform mixing of chemically distinct polymers within a hydrated micelle core. This allows us to observe how the charged block length of AB polyelectrolytes enhances or inhibits the ability of AC polyelectrolytes to contribute to the core size as a function of charged block length. To better understand this phenomenon, we examined the relationship between set block length and core growth for different polyelectrolyte chemistries.

**Figure 4:**
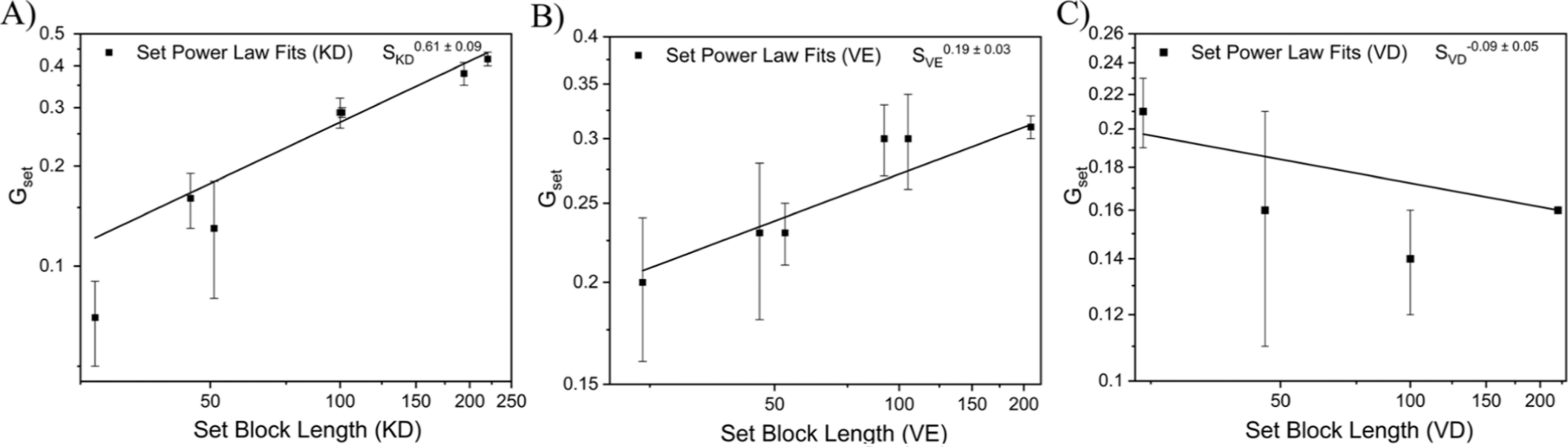
Set power law fits vs set block length for core radius from sets of data found in Figure 3A,B,D,E,G,H. Polyelectrolyte pairings of PEO-*b*-P*L*K and PEO-*b*-P(d,l)D, PEO-*b*-PVB and PEO-*b*-P(d,l)E, and PEO-*b*-PVB and PEO-*b*-P(d,l)D are denoted with KD, VE, and VD, respectively. A power law fit was applied to each data set. Sets from Figure 3 with fewer than three data points were excluded from this analysis. Error bars represent standard error.

To develop a systematic understanding of the effect of polyelectrolyte chemistry on interdependent PCM growth, we examined the relationship between the scaling exponents of each data set in Figure 3 and set block length. Fitting the combined data in Figure 4 allows us to extract an interdependent growth relationship (IGR) that serves to quantify how the set block length influences PCM core growth between sets (e.g., K100 vs K200) and how polyelectrolyte chemistry influences across polyelectrolyte pairings (e.g., KD vs VE). For example, the positive relationship between PCM growth per set of micelles (*G*_set_) and set block length for KD micelles suggests that if one increased the block length held constant from K200 to K300 and examined a series of PCMs formed from pairing K300 with D50-D200, one should expect the scaling exponent of that new data set to follow the IGR for KD of *S*_KD_^0.61±0.09^ (Figure 4A). The new relationship between set block length and core growth for the K300 set should grow with PRD block length (*N*_PRD_) by approximately, *R*_core_ ∝ *N*_PRD_^0.49±0.09^ assuming no significant morphological transition occurs as a result of the longer K block. In addition, we can use the magnitude of the IGRs of KD (*S*_KD_^0.61 ± 0.09^) and VE (*S*_VE_^0.19 ± 0.03^) to conclude that the effect of set block length is about three times more pronounced in KD polyelectrolyte pairings than in VE pairings. Interestingly, the IGR of VD micelles is slightly negative at *S*_VD_^–0.09 ± 0.05^, contrary to the positive values of KD and VE IGRs, suggesting there is a wide range of potential IGRs that are extremely sensitive to polyelectrolyte chemistry.

The behavior of different polyelectrolyte pairings on AB + AC micelle core growth stands in stark contrast to behavior observed in AB + C systems, where the influence of block length on micelle features can be predicted with a high degree of certainty independently of the polyelectrolyte chemistry.^43^ It remains unclear which chemical features of the different polyelectrolyte pairs change the growth rates of the PCM cores a priori. If one were to conclude that more hydrophilic polyelectrolyte chains promoted larger PCM cores from comparing the IGRs of the KD and VE system, one would then expect that the IGR of the VD system would be a positive value larger than that of the VE system, due to the marginally more hydrophilic backbone of the P(d,l)D chain relative to P*L*E. However, Figure 4 shows that not only is the IGR for VD smaller than the VE system, it is the only polyelectrolyte pair with a negative IGR. These trends highlight the pronounced difference in growth behavior between S-PCM and D-PCM cores.

### Ion Pair Aggregation Number

In an attempt to understand the origin of the difference between single and D-PCM core growth, we examined aggregation number evolution as a function of constant block length. The aggregation number represents the number of polymers associating within each nanoparticle. This quantity can be calculated from the forward scattering intensity of SAXS experiments, as described in the Supporting Information (Table S2). Calculating aggregation numbers in our systems requires us to assume that the cores of our micelles are charge neutral and that essentially all polyelectrolytes in our system contribute to micelle formation. These assumptions are reinforced by the typically low critical micelle concentration for PCMs^20^ and the neutral zeta potential measurements in our system (Figure S5). Using these assumptions, Marras et al.^43^ derived the following expression
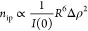
1where *n*_ip_ is the number of ion pairs per micelle, *R* is the core radius of the PCM, *I*(0) is the forward scattering intensity from SAXS, and Δρ is the calculated scattering contrast between polymer and solvent (Table S2).

**Figure 5:**
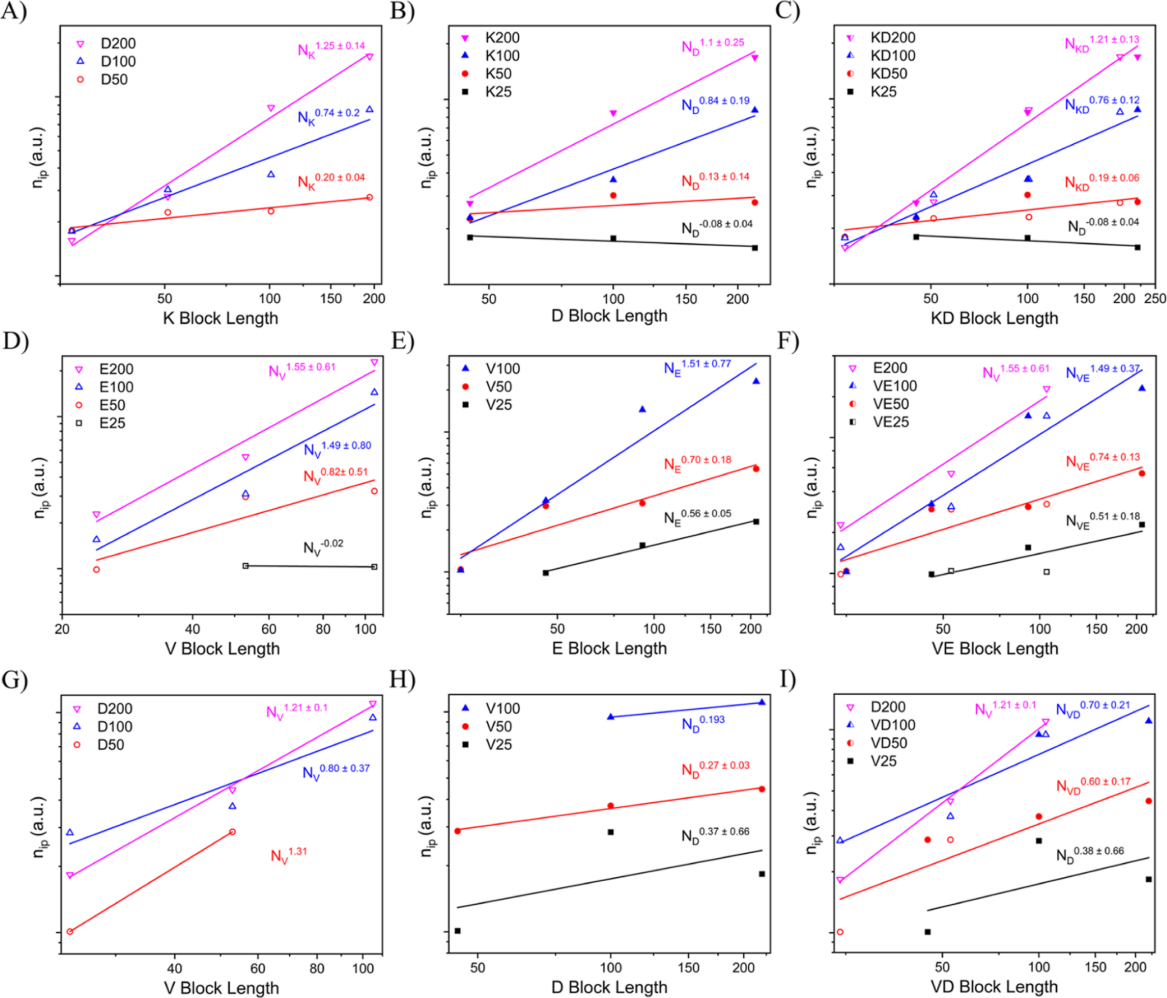
Aggregation number vs block length determined by fitting *I*(0) SAXS data and plotting the results against charged block length. A power law fit was applied to each data set, which are divided into separate colors/symbols. Every diblock polyelectrolyte has a neutral PEO block length of ∼5k. Charged block length names are abbreviated from P*L*K to K, PVB to V, P(d,l)D to D, and P(d,l)E to E for brevity. Across all datasets the datapoints/fits are represented by colors, with black representing DP ∼25, red symbols representing DP ∼50, blue symbols representing DP ∼100 and pink symbols representing DP ∼200. Open datapoints signify an anionic frame of reference, where the data are grouped into sets of constant anionic block length and plotted against varying cationic block length. Filled data signify a cationic frame of reference, where data are grouped into sets of constant cationic block length and plotted against varying anionic block lengths. Half-filled data signify a composite dataset where both anionic and cationic frames of reference are represented in the same color and are fit together. (A–C) Show the KD pairing. (A,B) show the distinct frames of reference and in (C) both datasets are overlaid to remove any preference for one diblock when fitting the results. (D–F) and (G–I) repeat the same graphing pattern for VE and VD polyelectrolyte pairings.

**Figure 6:**
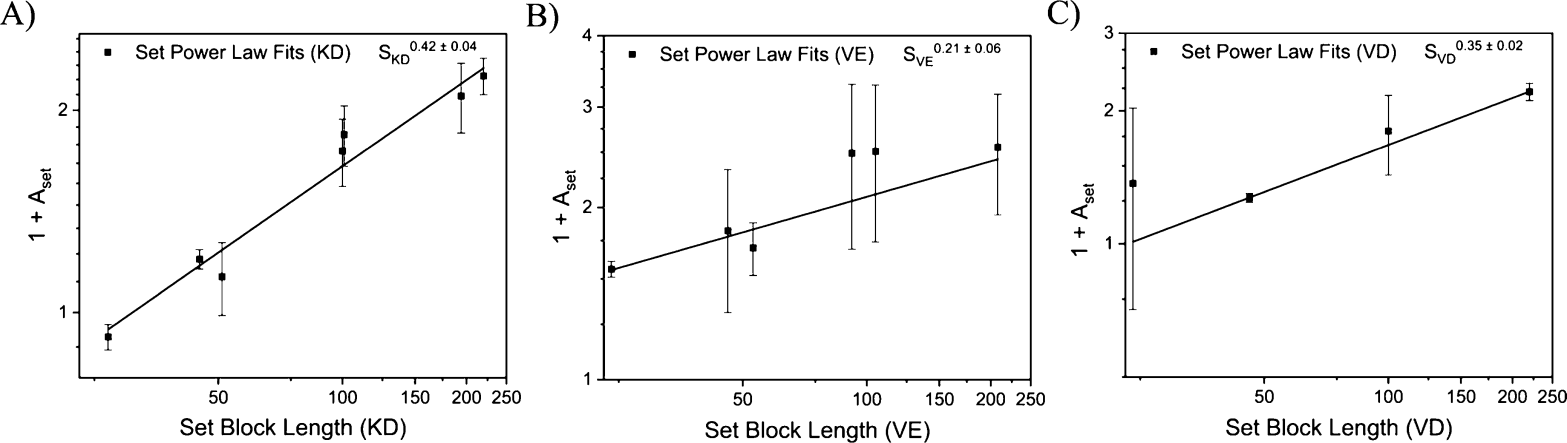
Set block length vs 1 + *A*_set_ where *A*_set_ is defined as the fits of ion pair aggregation number vs charged block length for each set from Figure 5A,B,D,E,G,H. Polyelectrolyte pairings of PEO-*b*-P*L*K and PEO-*b*-P(d,l)D, PEO-*b*-PVB and PEO-*b*-P(d,l)E, and PEO-*b*-PVB and PEO-*b*-P(d,l)D are denoted with KD, VE, and VD, respectively. A power law fit was applied to each data set. Sets from Figure 5 with fewer than three data points were excluded from this analysis. Error bars represent standard error.

This ion pair aggregation number (*n*_ip_) data was visualized using the same naming and color conventions as Figure 3. The *n*_ip_ increases with larger block length in most samples. Much like the data in Figure 3, the different sets in Figure 5 cannot be normalized to a single predictive curve because the slope of each set shows a systematic dependence on set block length, increasing with set block length. The dependence of the data set fits on set block length gives an interdependent monomeric aggregation number relationship (IAR) for each polyelectrolyte chemistry (Figure 6). Interestingly, there is no straightforward trend between the IAR and the IGR for each chemistry. One would expect that if density was held constant, the growth of aggregation number and core size should be related such that the trends between IAR and IGR were similar. This led us to investigate the relationship of aggregation number and core growth through the framework of monomer density within the PCM core.

### Core Density

If we consider the PCM core to be a homogenously distributed sphere of ion pairs with no higher order structure, then PCM core density should be related to the size and number of ion pairs within the core. Figures 3–6 suggest that the core radius (*R*) and ion pair aggregation number (*n*_ip_) do not grow with the *n*_ip_ ∼ *R*^3^ relationship one would expect if the monomer density within the PCM core were to remain constant. To understand how these trends relate to each other, we sought to create a variable *D*_set_ to represent the change in micelle density over each data set
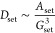
2here, *A*_set_ is the power law fit value for *n*_ip_ within the core for each PCM set in Figure 5, and *G*_set_ is the PCM core radius fit value for each set in Figure 3 where *R*^3^ ∼ PCM core volume. The value of *D*_set_ describes how the density within the core of a micelle changes over a single data set. A constant *D*_set_ across set block lengths would mean the extent that PCM core density changes PCM core density consistently across each set of micelles for that polyelectrolyte pair. Fitting the data allows us to extract an interdependent density relationship (IDR) that can be used to compare how the density changes evolve as set block length grows for each chemistry explored here (Figure S5). Unfortunately, the calculations for *D*_set_ propagate too much error for us to make definitive conclusions between different polyelectrolyte chemistries using the data in Figure S5.

While it is not possible to make definitive conclusions from the values of *D*_set_ due to error propagation, there is a consistent trend in the data whereby polyelectrolyte pairs with the largest IGRs have the lowest IDRs (Table 1). This suggests that if the core density growth were constant for every set block length, the effect of block length on core size would be quite large. However, if core density depends on block length, the relationship between block length and core size can decrease or even become negative. On a physical level, this relationship between core growth and density change could be explained by the molecular rearrangements required to simultaneously accommodate charge neutralization and strict core–shell separation in mismatched micelles. Greater understanding of how polyelectrolyte chemistry affects micelle core density could provide a route to predictive understanding of the interdependent micelle behavior of more general AB + AC systems. More meaningful exploration of this mechanism could perhaps be achieved through simulations and modeling.

**Table 1:** Summary of Interdependent Relationships for Core Growth, Aggregation Number, and Core Density^a^

polyelectrolyte pair	IGR	IAR	IDR*
KD	0.61 ± 0.09	0.42 ± 0.04	0.17 ± 0.02
VE	0.19 ± 0.03	0.21 ± 0.06	0.26 ± 0.07
VD	–0.9 ± 0.05	0.35 ± 0.02	0.47 ± 0.05

a All interdependent relationships are power law fits obtained from Figures 4, 6 and S5. *Some datapoints within the trendlines for IDR have large error propagation from eq 1. IDR is, therefore, more suggestive than conclusive and is tabulated here for ease of comparison with IGR.

### Comparison between Double and Single Diblock PCMs

In preceding sections, we detailed how D-PCMs, unlike S-PCMs, show a variety of interdependent structure–property relationships that cannot be simplified into a unifying scaling law. It is well known from decades of experiments that D-PCMs tend to have smaller core sizes and polymer aggregation numbers than S-PCMs of similar composition. However, little is known about how the trends in S-PCM and D-PCM structure–property relationships differ quantitatively. In Table 2, we compare the scaling laws derived by Marras et al. for S-PCMs and compare them to the range of growth behaviors observed in this work for D-PCMs (Tables S3–S5).^41^ Interestingly, it seems that not only do D-PCMs have smaller core sizes than S-PCMs but they also grow at a slower rate as a function of charged block length. As one would expect, the aggregation number growth is also smaller for the D-PCMs, although the largest values fall within error of S-PCMs. It is difficult to decipher if the density values differ from those of S-PCMs due to the large error in the scaling law for the ion pair density. Taken together, the results in Table 2 suggest that D-PCM geometries not only reduce the size and ion pair aggregation number relative to S-PCMs but the D-PCM configuration also restricts the growth of these structural features as a function of charged block length. The differences between S-PCMs and D-PCMs could be attributed to the attachment of corona-forming blocks to every core forming block in the case of D-PCMs. Presumably, this leads to D-PCMs being less able to accommodate the competing demands of core packing, charge matching and core–shell segregation over ranges of varying chain lengths than S-PCMs. Importantly, the comparisons in Table 2 cannot be easily generalized to chemistries beyond those explored in this work.

**Table 2:** Comparison of Structure–Property Relationships for S-PCMs and D-PCM Cores^a^

	S-PCMs (∝*N*_B_)	D-PCMs (∝*N*_B/C_) min	D-PCMs (∝*N*_B/C_) max
*R*_core_	0.73 ± 0.11	0.07 ± 0.02	0.42 ± 0.02
*n*_ip_	2.37 ± 0.41	–0.08 ± 0.04	1.55 ± 0.61
Φ_ip_	0.18 ± 0.74	–0.29 ± 0.10	0.73 ± 0.21

a Since single diblock scaling laws describe data from multiple discrete sets of PCMs, errors for single diblock PCM scaling relationships are standard deviations, whereas the errors for single diblocks are standard errors.

## Conclusions

Our results demonstrate that D-PCMs can be formed from a wide range of mismatched charged block lengths using a variety of chemistries; something that was previously thought to be unattainable for PCMs. This work analyzes structures formed by pairs of polyelectrolytes with block length mismatches of up to ∼1000% (K25D200), moving far beyond the <10% charged block length difference usually chosen for PEC self-assembly. We see a correlation between PCM core radius and charged block length, as expected, and uncover a unique relationship between these two parameters. Instead of constant scaling as a function of the core forming block length, we observe an interdependent growth mechanism whereby one charged block length influences the ability of the other charged block length to contribute to core growth. Examining the relationship between power law fits and block lengths revealed interdependent growth behaviors that estimate PCM core size as each charged block length is varied for PCMs of the same chemistry. These behaviors also illustrate the effect of block length on PCM core radius between different PCM chemistries. This unique interdependent behavior extends to the aggregation number of these micelles with a similar trend. Finally, we use the combination of aggregation number and growth patterns to estimate a relationship for core density. We postulate that the effects seen on changes in core size growth could be an indirect consequence of changes in core density but are unable to verify this experimentally due to propagated error. We encourage contributions using simulations and theory to gain further mechanistic insights and understanding to this open question. Finally, we compare the scaling relationships observed for S-PCMs to the interdependent growth behaviors extracted for D-PCMs and find that *R*_core_ and *n*_ip_ grow at a reduced rate in D-PCMs compared to S-PCMs.

Our results have broad implications for the design of nanostructured PEC materials. We show that processing conditions can overcome the limitations of chain length recognition, and that introducing mismatched block lengths in a PEC nanostructure can lead to unique behavior within PCM cores, which we quantify here. The implications of these findings raise questions about how mismatched charged block lengths affect the mechanical structure of PEC gels, the chain exchange of PCMs and the permeability and delivery efficacy of PEC polymersomes, as the density and size of PEC domains is central to all these phenomena. Further work on the role of design parameters like neutral block length and polymer concentration on mismatched D-PCMs could serve to deepen our understanding of structure–property relationships within D-PCMs and charged assemblies more broadly.

## Experimental Section

### Materials

Poly(ethylene oxide)-*b*-poly(l-lysine hydrochloride) (PEO-*b*-PLK), poly(ethylene oxide)-*b*-poly(d,l glutamic acid) (PEO-*b*-P(d,l)E), and poly(ethylene oxide)-*b*-poly(d,l aspartic acid) (PEO-*b*-P(d,l)D) were purchased from Alamanda Polymers and used as received. Degree of polymerization provided from the manufacturer is used in all calculations (Table S5). Poly(ethylene oxide)-*b*-((vinylbenzyl) trimethylammonium chloride) (PEO-*b*-PVBTMA) were synthesized via reversible addition–fragmentation chain-transfer (RAFT) polymerization of (vinylbenzyl) trimethylammonium chloride monomer from 5000 g/mol PEO trithiocarbonyl macro-CTA according to previous conditions.^48^

### Polyelectrolyte Complex Micelle Preparation

Stock solutions were prepared by mixing the desired amount of polymer and water, vortexing for 1 min, and sonicating for 5 min, per the instructions provided by Alamanda Polymers. Stock solutions of polymer, salt, and *N*-(2-hydroxyethyl)piperazine-*N′*-ethanesulfonic acid (HEPES) were mixed such that the final solutions were 5 M NaCl, 2mM charge concentration for each polymer, and 10 mM HEPES buffer (pH 7.4). These high salt mixtures were then dialyzed over the course of days until a final concentration of 50 mM NaCl and 10 mM HEPES in water was reached, following our published protocols.^42^ These solutions were stored at 4 °C and allowed to equilibrate for at least 2 h at room temperature before any experiments were performed.

### Dynamic Light Scattering

DLS measurements were performed on a Brookhaven Instruments BI-200SM Research Goniometer System with an incident wavelength of 637 nm and a 90° scattering angle. Dust-free decalin was used as a bath to match the refractive index of the glass sample tubes. The Stokes–Einstein relationship was used to calculate the hydrodynamic radius under Brownian diffusion. Autocorrelation functions were analyzed via cumulant analysis in MATLAB.

### Zeta Potential

ζ-potential measurements were performed on a Wyatt Mobius Dynamic/Electrophoretic Light Scattering instrument using Dynamics (Version 7.4.072) software. Micelle samples were loaded into a quartz cuvette for ζ-potential measurements. A voltage amplitude of 2 V was applied at a frequency of 10 Hz and collected for 15 s at 25 °C five separate times. Three scans were averaged together and converted to ζ-potentials via the Smoluchowski equation.

### Small-Angle X-Ray Scattering

SAXS measurements were made at Argonne National Laboratory’s Advanced Photon Source at beamline 12-ID-B. To minimize radiation damage, SAXS samples were prepared at 1% glycerol by volume. Micelles were irradiated in a thin-walled glass capillary flow cell with a 14 keV photon energy. Data reduction, background subtraction, and data fitting were performed using the Irena plugin package within Igor Pro as described in ref (40). Raw SAXS data will be uploaded to the Materials Data Facility.^49^

### Transmission Electron Microscopy

To obtain cryo-TEM images, 3.5 μL of PCM sample was blotted onto a plasma cleaned Quantifoil copper grid 200 mesh 1.2/1.3, blotted for 1 s, and plunged into liquid ethane. Images were taken on a Thermo Titan Krios G3i at 300 kV at 81 000× with a Gatan K3 direct detector.

## Supporting Information Available

TEM, DLS, and SAXS fitting results, tables of power law fits for micelle sets, polymer characterization, and and zeta potential (PDF)
