# Supplementary material for: Effect of Charged Block Length Mismatch on Double Diblock Polyelectrolyte Complex Micelle Cores
Source: Macromolecules. 2023 Jul 6;56(14):5557–66. doi: 10.1021/acs.macromol.3c00555 (PMC10373519; doi:10.1021/acs.macromol.3c00555)
Supplement: Supplementary file 1 — ma3c00555_si_001.pdf [file ma3c00555_si_001.pdf]

Supplemental Information for

Effect of Charged Block Length Mismatch on Double Diblock Polyelectrolyte Complex Micelle Cores

*Kaden C. Stevens[1], Alexander E. Marras[2,3], Trinity R. Campagna[1], Jeffrey M. Ting[4], Matthew V. Tirrell[*1]*

[1]Pritzker School of Molecular Engineering, The University of Chicago, Chicago, Illinois 60637, USA
[2]Walker Department of Mechanical Engineering, The University of Texas at Austin, Austin, Texas, 78712, USA
[3]Texas Materials Institute, The University of Texas at Austin, Austin, Texas, 78712, USA
[4]Nanite Inc., Boston, Massachusetts 02109, USA

Table of Contents

S1. Scattering Length Density 2
S2. Transmission Electron Microscopy 3
S3. Fitting Results and SAXS Profiles 4
S4. Polymer Characterization 8
S5. Supplemental Fitting Results 9
S6. Zeta Potential 11
S7. SI References 12

S1. Scattering Length Density

Scattering length density (SLD, $\boldsymbol{\rho}$) was calculated in Origin using the built-in contrast calculator within the Irena plugin bundle for Igor by inputting the chemical composition for each material and solvent. Contrast values **($\Delta\boldsymbol{\rho}$)** in Table S1 are proportional to SAXS scattering intensity. The values in table S1 support our assumption that PEG contributes minimally to our SAXS data and that the models fit to our data only represent PCM cores.

Table S1. The built-in density calculator from the Irena scattering plugin package within Igor was used to obtain SLD and contrast values, which are used below in the derivation for n_{ip}.

Material	SLD ($\boldsymbol{\rho}$) (10^{10} cm^{-2})	Contrast $(\Delta\boldsymbol{\rho})^2$ $(10^{10}\ cm^{-2})^2$
**Lysine**	12.71	10.82
**VBTMA**	12.11	7.24
**Aspartic Acid**	12.62	10.24
**Glutamic Acid**	12.34	8.53
**PEO**	10.36	0.88
**Solvent**	9.42	0.00

S2. Transmission Electron Microscopy

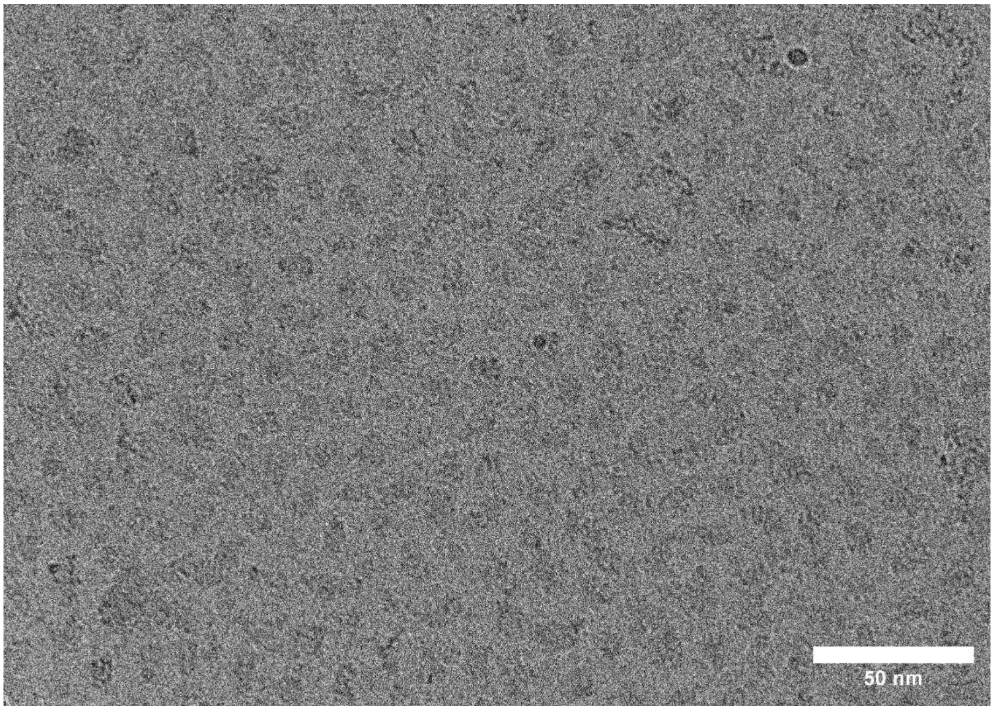

Figure S1. Cryo TEM of K200D50.

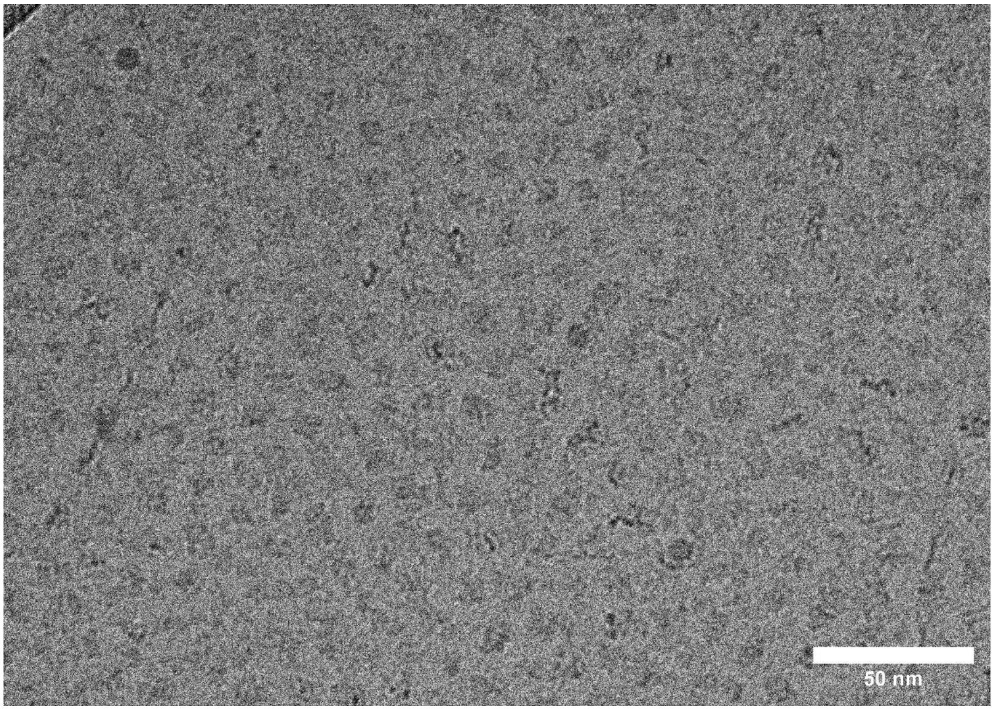

Figure S2. Cryo TEM of V50E50

S3. SAXS Fitting and Fitting Results

In Table S2, we tabulate results for each PCM (Figures 2-6) are tabulated in Table S2. PCM core size and aggregation number were characterized via SAXS and modeled in the Irena[1] plugin for Igor Pro according to previous methods.[2, 3] DLS data were fit using a cumulant fitting algorithm. Corona thickness was calculated as $H=R_h-R_{core}$.

Table S2. Fitting results for SAXS and DLS data.

PCM components	Core structure from SAXS				Hydrodynamic size from DLS		
**Sample Label**	**Intensity (q=0)**	**R_{core}: Mean Radius (nm)**	**PDI**	**Aspect Ratio**	**R_h: Mean Radius (nm)**	**PDI**	**H: Corona Thickness (nm)**
K25D50	0.157	9.3	0.05	0.53	12.5	0.09	3.2
K25D100	0.255	10.0	0.04	0.60	14.3	0.05	4.3
K25D200	0.344	10.3	0.06	0.65	15.1	0.05	4.7
K50D50	0.415	11.3	0.03	0.61	18.0	0.03	6.6
K50D100	0.919	13.6	0.01	0.69	22.4	0.07	8.8
K50D200	1.289	14.2	0.01	0.70	18.5	0.02	4.3
K100D50	0.583	12.0	0.03	0.61	22.2	0.25	10.2
K100D100	1.546	15.3	0.02	0.88	21.8	0.03	6.5
K100D200	2.363	19.0	0.01	0.77	24.7	0.02	5.7
K200D50	0.803	13.1	0.03	0.62	57.8	0.42	44.7
K200D100	2.274	18.8	0.01	0.72	24.6	0.03	5.8
K200D200	5.525	24.4	0.01	0.77	30.5	0.05	6.1
V25E25	-	-	-	-	-	-	-
V25E50	0.192	9.6	0.09	0.62	42.0	0.85	32.4
V25E100	0.631	11.6	0.05	0.64	16.8	0.23	4.2
V25E200	0.551	13.1	0.02	0.65	17.0	0.23	3.9
V50E25	0.227	9.9	0.08	0.54	20.6	0.54	10.7
V50E50	0.283	12.3	0.14	0.62	17.6	0.17	5.4
V50E100	0.633	14.1	0.10	0.73	19.5	0.07	5.4
V50E200	0.89	16.4	0.07	0.75	22.3	0.06	5.9
V100E25	0.227	9.9	0.10	0.62	15.2	0.19	5.3
V100E50	0.494	13.6	0.12	0.72	19.1	0.07	5.5
V100E100	0.549	17.8	0.16	0.74	24.5	0.12	6.7
V100E200	0.831	20.6	0.12	0.72	25.9	0.05	5.3
V25D50	0.183	9.2	0.09	0.67	19.7	0.50	10.5
V25D100	0.35	12.2	0.02	0.63	17.9	0.21	5.7
V25D200	0.873	13.2	0.01	0.67	19.9	0.40	6.7

V50D50	0.231	11.4	0.12	0.53	16.9	0.15	5.5
V50D100	0.604	14.0	0.07	0.75	21.8	0.11	7.8
V50D200	0.768	15.0	0.06	0.75	22.6	0.11	7.6
V100D50	-	-	-	-	-	-	-
V100D100	0.359	14.9	0.25	0.81	25.9	0.18	11.0
V100D200	0.595	16.7	0.26	0.89	25.1	0.15	8.4

Table S3. Example of core-shell model fitting of SAXS data.

Sample Label	R_{core}: Mean Radius (nm)	Shell Thickness (nm)	Total Radius (nm)	R_h: Mean Radius (nm)
V25D50	6.6	1.6	8.2	19.7
V25D100	7.6	3.5	11.1	17.9
V25D200	8.9	3.7	12.6	19.9
V50D50	7.7	1.6	9.3	16.9
V50D100	10.4	3.9	14.3	21.8
V50D200	11.7	3.5	15.2	22.6
V100D50	-	-	-	-
V100D100	10.8	4.6	15.4	25.9
V100D200	13.6	5.5	19.1	25.1

Due to large differences in scattering contrast between these PCM core and corona blocks (Table S1), a spheroid form factor was used to fit SAXS data. While this follows convention for these materials, some PCMs have been fit with a core-shell model in the past. Due to this discrepancy, we separately fit a representative subset of our SAXS data with a core-shell form factor, with results shown in Figure 3. The fitting results shown here demonstrate the poor alignment of sizes obtained via DLS and core-shell fitting. This is one of the reasons we believe core-shell fitting protocols fail to accurately describe our data. Intuitively, fitting our data via a core-shell model would seem appropriate, as our materials do form core-shell particles. However, we believe that the faint scattering of PEO is masked by the prominent scattering from the core since the scattering contrast (Table S1) and density of the core-forming blocks is much greater than that of PEO. Our position is reinforced by Cryo-TEM, which also relies on electron density to obtain contrast and fails to show core-shell geometries for any PCMs we have studied due to weak electron density contrast between PEO and water. Furthermore, our group has previously observed divergent structural trends in the size estimations from SAXS and DLS in S-PCMs. For example, previous studies from our group have shown that as PEO block MW (N_A) is increased from 5kDa to 20kDa, core size estimated via spheroidal fitting of SAXS decrease slightly as $R_{core} \propto N_A^{-0.17 \pm 0.04}$ whereas the hydrodynamic radius (R_h) estimated through DLS analysis grew as $R_h \propto N_A^{0.17 \pm 0.06}$.[2] This supports the hypothesis that PEO coronas are not noticeable via SAXS in the presence of high contrast micelle cores. The presence of this and other divergent structural trends for PCMs obtained through DLS and SAXS directly contradicts the idea that the two methods are both modeling the entire PCM, further reinforcing our position that the core is the dominant scattering body in SAXS of PCMs.

Table S4. Power law fits for set block length vs core size for KD, VE and VD micelle.

Set Label (KD)	Fit	Set Label (VE)	Fit	Set Label (VD)	Fit
K25(D)	0.07 ± 0.02	V25(E)	0.20 ± 0.04	V25(D)	0.21 ± 0.02
K50(D)	0.13 ± 0.05	V50(E)	0.23 ± 0.02	V50(D)	0.1 ± 0.05
K100(D)	0.29 ± 0.01	V100(E)	0.30 ± 0.04	V100(D)	0.13
K200(D)	0.38 ± 0.03	E25(V)	0.08	D50(V)	0.27
D50(K)	0.16 ± 0.03	E50(V)	0.23 ± 0.05	D100(V)	0.14 ± 0.02
D100(K)	0.29 ± 0.03	E100(V)	0.30 ± 0.03	D200(V)	0.16 ± 5E-4
D200(K)	0.42 ± 0.02	E200(V)	0.31 ± 0.01	VD50	0.21 ± 0.03
KD50	0.16 ± 0.03	VE25	0.2 ± 0.04	VD100	0.14 ± 0.01
KD100	0.28 ± 0.02	VE50	0.23 ± 0.02	S_{rad}(VD)	-0.9 ± 0.05
KD200	0.40 ± 0.02	VE100	0.29 ± 0.02		
S_{rad}(KD)	0.61 ± 0.09	S_{rad}(VE)	0.19 ± 0.03		

Table S5. Power law fits for set block length vs ion pair aggregation number from SAXS data.

Set Label (KD)	Fit	Set Label (VE)	Fit	Set Label (VD)	Fit
K25(D)	-0.08 ± 0.04	V25(E)	0.56 ± 0.05	V25(D)	0.37 ± 0.66
K50(D)	0.13 ± 0.14	V50(E)	0.70 ± 0.18	V50(D)	0.27 ± 0.03
K100(D)	0.84 ± 0.19	V100(E)	1.51 ± 0.77	V100(D)	0.193
K200(D)	1.1 ± 0.25	E25(V)	-0.02	D50(V)	1.31
D50(K)	0.2 ± 0.04	E50(V)	0.82 ± 0.51	D100(V)	0.80 ± 0.37
D100(K)	0.74 ± 0.2	E100(V)	1.49 ± 0.80	D200(V)	1.21 ± 0.10
D200(K)	1.25 ± 0.04	E200(V)	1.55 ± 0.61	VD50	0.60 ± 0.17
KD50	0.19 ± 0.06	VE25	0.51 ± 0.18	VD100	0.70 ± 0.21
KD100	0.76 ± 0.12	VE50	0.74 ± 0.13	S_{agg}(VD)	0.35 ± 0.02
KD200	1.21 ± 0.13	VE100	1.49 ± 0.37		
S_{agg}(KD)	0.42 ± 0.04	S_{agg}(VE)	0.21 ± 0.06		

Table S6. Power law fits for set block length vs ion pair density calculated from table S3 and S4.

Set Label (KD)	Fit	Set Label (VE)	Fit	Set Label (VD)	Fit
K25(D)	-0.29 ± 0.1	V25(E)	-0.04 ± 0.17	V25(D)	-0.26 ± 0.72
K50(D)	-0.26 ± 0.29	V50(E)	0.01 ± 0.24	V50(D)	-0.21 ± 0.18
K100(D)	-0.03 ± 0.22	V100(E)	0.61 ± 0.89	V100(D)	-
K200(D)	-0.04 ± 0.34	E25(V)	-	D50(V)	-
D50(K)	-0.28 ± 0.04	E50(V)	0.13 ± 0.66	D100(V)	0.38 ± 0.43
D100(K)	-0.13 ± 0.29	E100(V)	0.59 ± 0.89	D200(V)	0.73 ± 0.10
D200(K)	-0.01 ± 0.1	E200(V)	0.62 ± 0.64	S_{den}(VD)	0.47 ± 0.05
S_{den}(KD)	0.17 ± 0.02	S_{den}(VE)	0.26 ± 0.07		

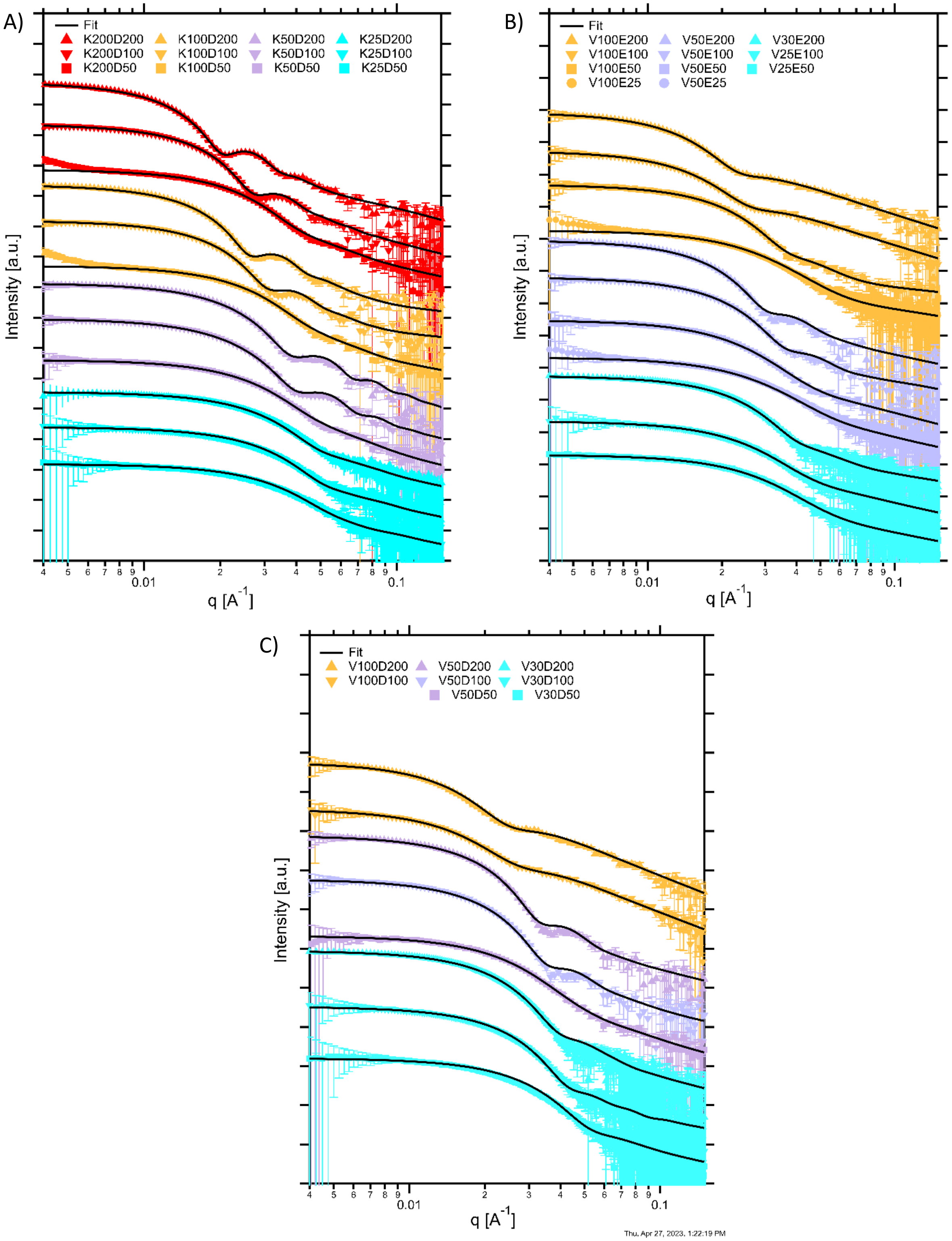

Figure S3. SAXS profiles and model fits for A) KD B) VE and C) VD polyelectrolyte pairings. Some data have been vertically offset for clarity.

S4. Polymer Characterization

Table S7. Polymer characterization data provided by Alamanda Polymers, INC.

Polymer	Charged DP	M_n of mPEG-NH_2 (kg/mol)	Mn by NMR (kg/mol)	PDI by GPC
K25	27	5.3	9.7	1.05
K50	51	4.6	13.0	1.09
K100	101	4.6	21.2	1.05
K200	194	4.6	36.5	1.07
D50	45	5.3	11.5	1.02
D100	100	4.6	18.3	1.04
D200	220	5.0	35.1	1.01
E25	25	5.0	8.7	N/A
E50	46	5.3	12.2	1.05
E100	92	5.1	19.0	1.02
E200	208	5.1	36.5	1.02

S5. Supplementary Fitting Results

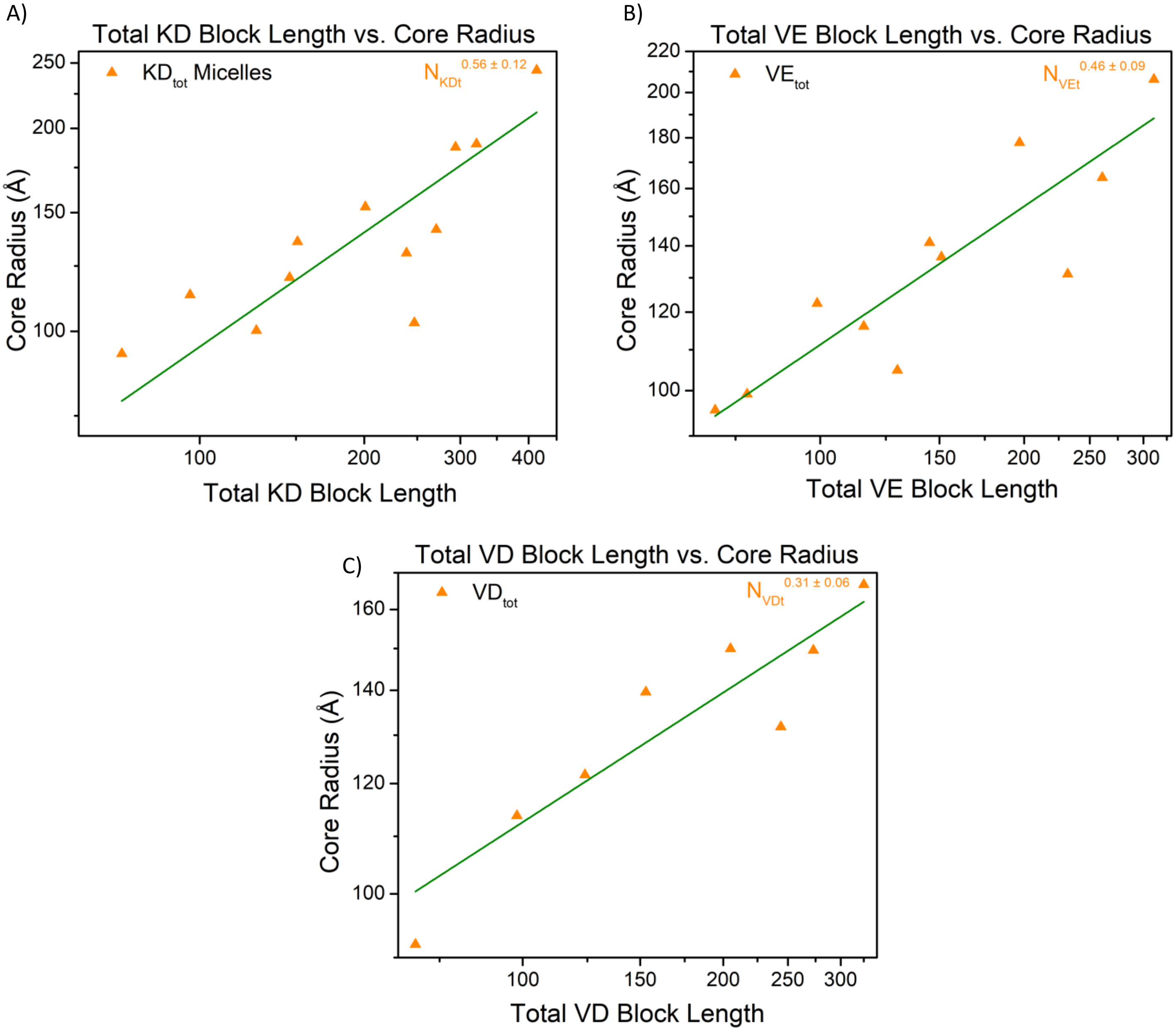

Figure S4. Total charged block length vs. core radius.

D)

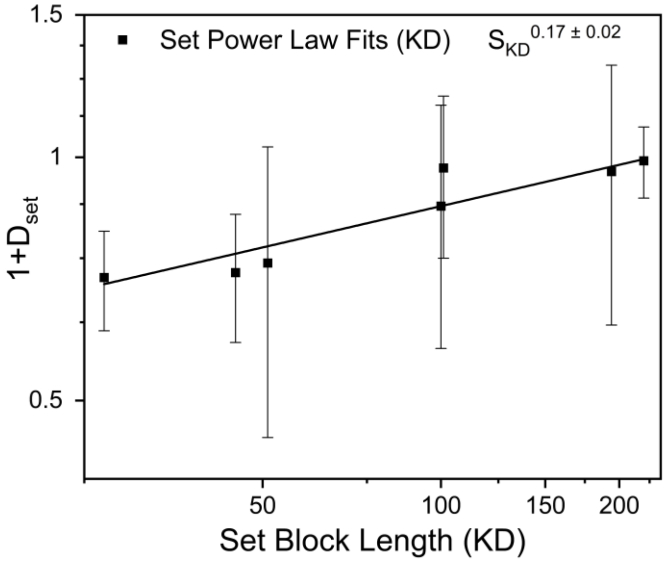

E)

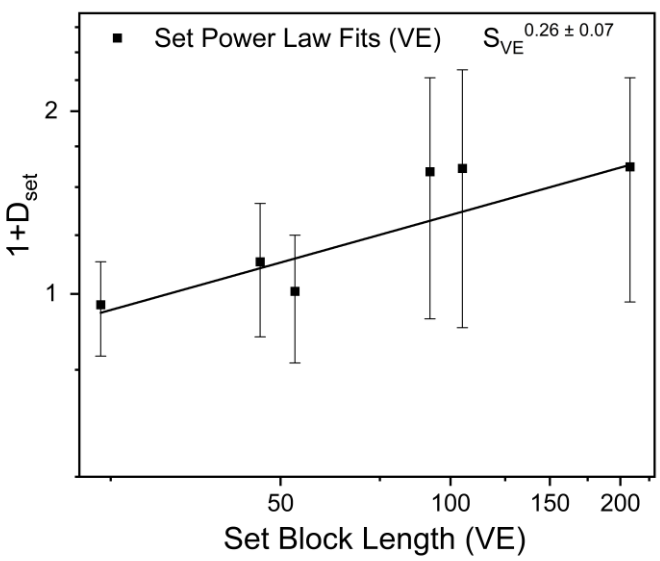

F)

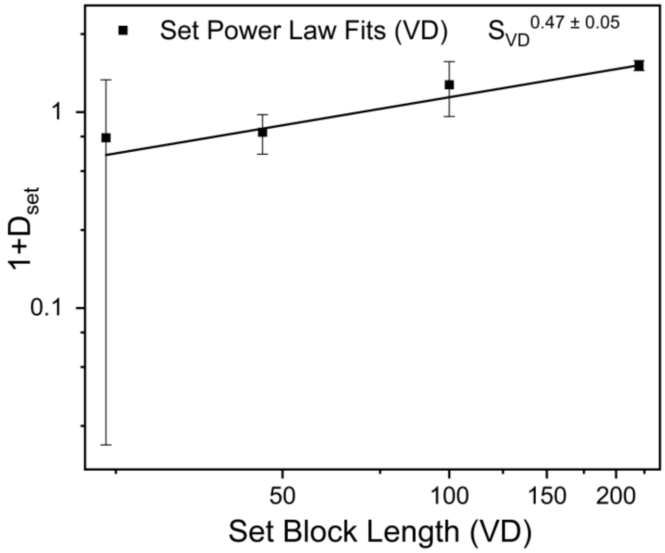

Figure S5. Set block length vs 1 + D_{set} where D_{set} is defined as $\frac{A_{set}}{G_{set}^{3}}$ where A_{set} is defined as the fits of ion pair aggregation number vs charged block length for each set from Fig 5 A,B,D,E,G and H and G_{set} is the fit of core radius from sets of data found in Fig. 3 A,B,D,E,G and H. Polyelectrolyte pairings of PEO-*b*-P*L*K and PEO-*b*-P(*D*,*L*)D, PEO-*b*-PVB and PEO-*b*-P(*D*,*L*)E and PEO-*b*-PVB and PEO-*b*-P(*D*,*L*)D are denoted with KD, VE, and VD, respectively. A power law fit was applied to each data set. Sets from Figure 5 with fewer than three data points were excluded from this analysis. Error bars represent standard error.

S6. Zeta Potential

A)

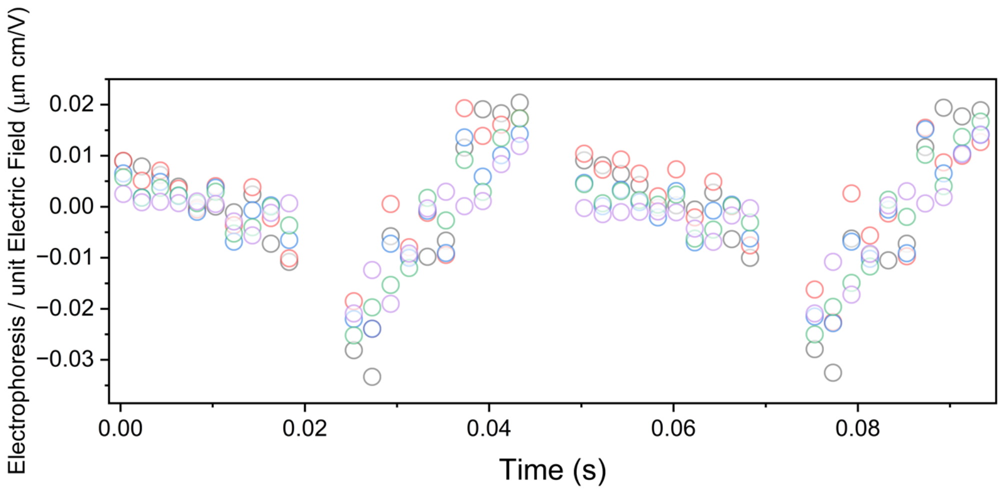

B)

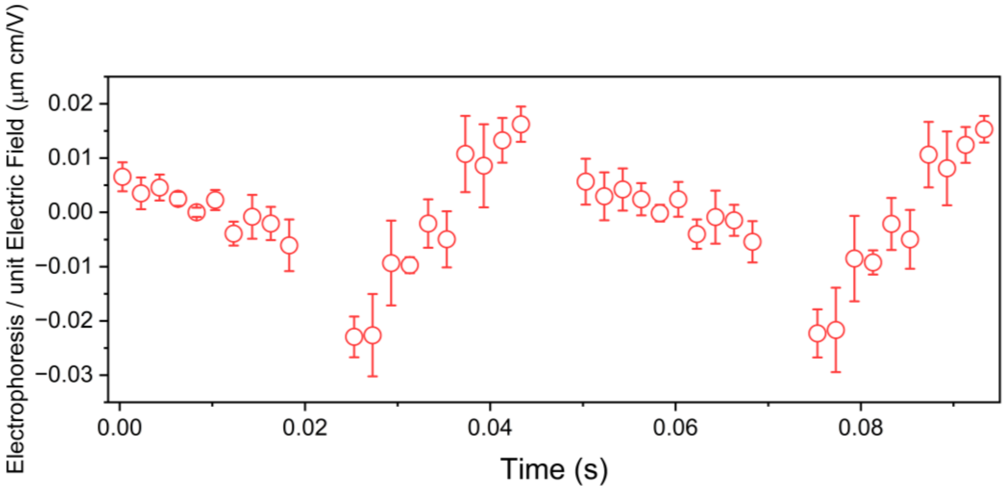

Figure S6. Representative mobility curve for V100E25 micelle. A) Raw data from 5 separate measurements. B) Averaged data and standard deviation.

Table S8. Zeta potential values. All values are averages and standard deviations from 5 measurements.

Micelle Name	Mobility (μm cm/s V)	Zeta Potential (mV)	Conductivity (mS/cm)	PALS Amplitude (mV)
V100E25	0.0 ± 0.05	0.00 ± 0.63	5.98 ± 0.08	78.7 ± 20.1
V100E50	$4\text{x}10^{-3}$ ± 0.03	0.09 ± 0.38	5.86 ± 0.04	97.12 ± 0.51
V100E100	$4\text{x}10^{-3}$ ± 0.11	0.06 ± 1.44	5.88 ± 0.08	109 ± 6.29
V100E200	$-2\text{x}10^{-3}$ ± 0.04	-0.03 ± 0.46	5.48 ± 0.05	167 ± 79.53

S7. SI References

1. Ilavsky, J.; Jemian, P. R. Irena: tool suite for modeling and analysis of small-angle scattering. *Journal of Applied Crystallography* **2009,** 42 (2), 347-353.

2. Marras, A. E.; Campagna, T. R.; Vieregg, J. R.; Tirrell, M. V. Physical property scaling relationships for polyelectrolyte complex micelles. *Macromolecules* **2021,** 54 (13), 6585-6594.

3. Marras, A. E.; Vieregg, J. R.; Tirrell, M. V. Assembly and Characterization of Polyelectrolyte Complex Micelles. *J Vis Exp* **2020,** (157), e60894 DOI: 10.3791/60894.
